# Improving Knowledge About Stroke Using Simulation Training

**DOI:** 10.7759/cureus.75143

**Published:** 2024-12-05

**Authors:** Tanvi Ambulkar, Prachi Ambulkar, Anamika Saha, Jasmesh Sandhu, Alisha Gurung, Chris Jacobs

**Affiliations:** 1 Medical Education, Great Western Hospitals NHS Foundation Trust, Swindon, GBR; 2 Critical Care, King's College Hospital NHS Foundation Trust, London, GBR; 3 Stroke Medicine, Great Western Hospitals NHS Foundation Trust, Swindon, GBR; 4 Urology, King's College Hospital NHS Foundation Trust, London, GBR; 5 Medicine, Great Western Hospitals NHS Foundation Trust, Swindon, GBR

**Keywords:** acute ischaemic stroke, ct scan head, door-to-needle time, mechanical thrombectomy (mt), medical student teaching, nihss score, reperfusion therapies, resident doctor, simulation in medical education, stroke thrombolysis

## Abstract

Background

Stroke is a medical emergency that is risk-stratified using a national scoring system called the National Institute of Health Stroke Scale (NIHSS). The management of an acute stroke necessitates prompt management and swift decision-making. Human factors were identified in the literature as the main rate-limiting step to improving door-to-needle (DTN) time. We felt it would be prudent to design a local stroke course implemented at Great Western Hospital Swindon that incorporates both traditional and simulation-based elements to improve theoretical knowledge and emulate real-life scenarios. The objective of this course was to improve practical application in the efficient assessment and management of stroke patients, as this is critical to delivering timely treatment with thrombolysis or thrombectomy.

Methods

Twenty-four medical professionals (medical students and resident doctors) participated in our course between November 2022 and July 2023. The domains assessed included understanding thrombolysis, understanding thrombectomy, confidence in performing NIHSS, and confidence in the assessment of stroke patients. The effectiveness of the stroke simulation course was assessed both quantitatively and qualitatively with pre- and post-course questionnaires.

Results

There was a significant improvement (p<0.05) in all four assessed domains. There was a significant increase (p=0.0003) in the mean difference of score 3.75 (95% CI: 2.43-5.07) in understanding thrombolysis. Similarly, understanding of thrombectomy was significantly improved (p=0.0002) with a mean difference in score of 3.4 (95% CI: 2.28-4.46). There was also a significant increase (p<0.0001) in confidence in completing NIHSS scoring by a mean of 4.33 (95% CI: 3.55-5.12). Lastly, there was a significant increase (p=0.0012) in the mean by 2.75 (95% CI: 1.51-3.99) in confidence in the assessment of stroke. Overall, 95.8% of the participants found the course at least good, if not very good or excellent, and 91.7% would recommend this course to others.

Conclusion

We found traditional and simulation-based training to be effective in improving understanding of thrombolysis, understanding of thrombectomy, confidence in NIHSS scoring, and confidence in the assessment of stroke patients. This study validates the effectiveness of our course in improving assessment and management in acute stroke patients. We infer that improvements in these domains coupled with simulation training focused on human factors (e.g., fatigue affecting decision-making or logistical issues such as delays in neuroimaging due to scanner availability) would achieve better DTN time in the participants of our course.

## Introduction

Stroke is the second-leading cause of mortality after ischaemic heart disease (IHD) worldwide [1] and the fourth highest in England and Wales, causing 5.1% of all deaths in 2022 (0.8% increase compared to 2021) [2]. Stroke is also known to be a risk factor in the development of dementia [3,4] and Alzheimer's [5], which was the number one cause of mortality, accounting for 11.5% of registered deaths in England and Wales in 2022 [2]. In the United Kingdom, approximately 1.3 million are living with a stroke at an estimated cost of £26 billion per year, including £8.6 billion for the National Health Service (NHS) and social care [6]. Although reperfusion therapy (intravenous thrombolysis and mechanical thrombectomy) has shown promise in managing ischaemic stroke [7,8], the mainstay of clinical outcomes ultimately relies on a narrow time window to intervene [9,10]. This suggests that effective clinical assessment and swift decision-making are essential to reduce mortality, improve quality of life after a stroke, and reduce financial burden.

The National Stroke Service Model 2021 recommends an ideal door-to-needle (DTN) time of 20 minutes [11]. Level one evidence suggests simulation training reduces DTN time by approximately 15 minutes [12]. This is critical as every minute in cerebral ischaemia can cause a loss of up to 1.9 million neurons, which can result in irreversible neurological damage [13]. Since the human factor is the main rate-limiting step [14], we have designed a course incorporating both traditional and simulation-based training to improve participant confidence. The primary outcomes are to aid medical professionals in making an efficient assessment of patients with a suspected stroke, improving confidence in the National Institute of Health Stroke Scale (NIHSS) scoring, improving understanding of thrombolysis and thrombectomy, and hence reducing DTN time. 

## Materials and methods

Course design and study parameters

The course design involved the circulation of a pre-reading booklet via email to participants prior to course attendance. The booklet encompassed information about the classification of stroke, NIHSS scoring, and clinical management of stroke. Participants subsequently attended an in-person course that involved reiteration of key points in the pre-reading material, as well as the opportunity to participate in simulated acute stroke scenarios. Quantitive feedback on self-reported performance on domains (understanding thrombolysis, understanding thrombectomy, confidence in performing NIHSS, and confidence in assessment of stroke patients) was measured on a Likert scale ranging from 1 to 10 (Appendix A). The difference between the mean score pre- and post-course was recorded to determine if there was significant improvement following course attendance (p<0.05). Qualitative analysis was obtained from a post-course primary survey (Appendix B). The data presented in all four domains was collected in line with the Standards for Quality Improvement Reporting Excellence (SQUIRE) 2.0 guidelines.

Sample size 

A sample size of 24 professionals, which comprised medical students and resident doctors, attended our course in a single centre (Great Western Hospital Swindon) between November 2022 and July 2023. However, only eight professionals were included in the analysis of four domains as advanced questionnaires and primary surveys independently assessing these domains were only introduced in the later stages of the course. We recognise one of the limitations is that this is an underpowered study.

Inclusion and exclusion criteria

Inclusion criteria for the participants included medical students (between the third to sixth year of medical school) and resident doctors (between the first foundation year and pre-registrar grades) who were primarily based at Great Western Hospital and had adequately completed a pre-course questionnaire. Exclusion criteria involved individuals who had pursued higher specialty training in neurology or stroke, candidates who had previously attended the stroke simulation program, and individuals from external hospital trusts or medical schools.

Statistical analysis

Data were tabulated in a Statistical Package for the Social Sciences (SPSS) so that the mean difference in score could be calculated. The Kolmogorov-Smirnov test was applied, which determined that data were normally (Gaussian) distributed in all four domains. Given our small sample size (<50 participants), we decided to perform a parametric dependent two-tailed T-test comparing data from pre- and post-course questionnaires. Subgroup analysis was further performed using a two-way ANOVA test. A value of p<0.05 was considered significant. The data was collected confidentially in accordance with Data Protection Laws and Caldicott Principles. 

## Results

Of eight participants, 75% were female and 25% were male. The mean age of medical students was <24 years, and resident doctors >24 years. To our knowledge, no graduate entry medical students were included. We had a balanced 1:1 ratio of medical students to resident doctors. The participants' demographic information is included in Table 1.

**Table 1:** Demographic characteristics of participants

Characteristic		n
Gender	Male	2
	Female	6
Medical professional	Medical student	4
	Resident doctor	4

**Figure 1:**
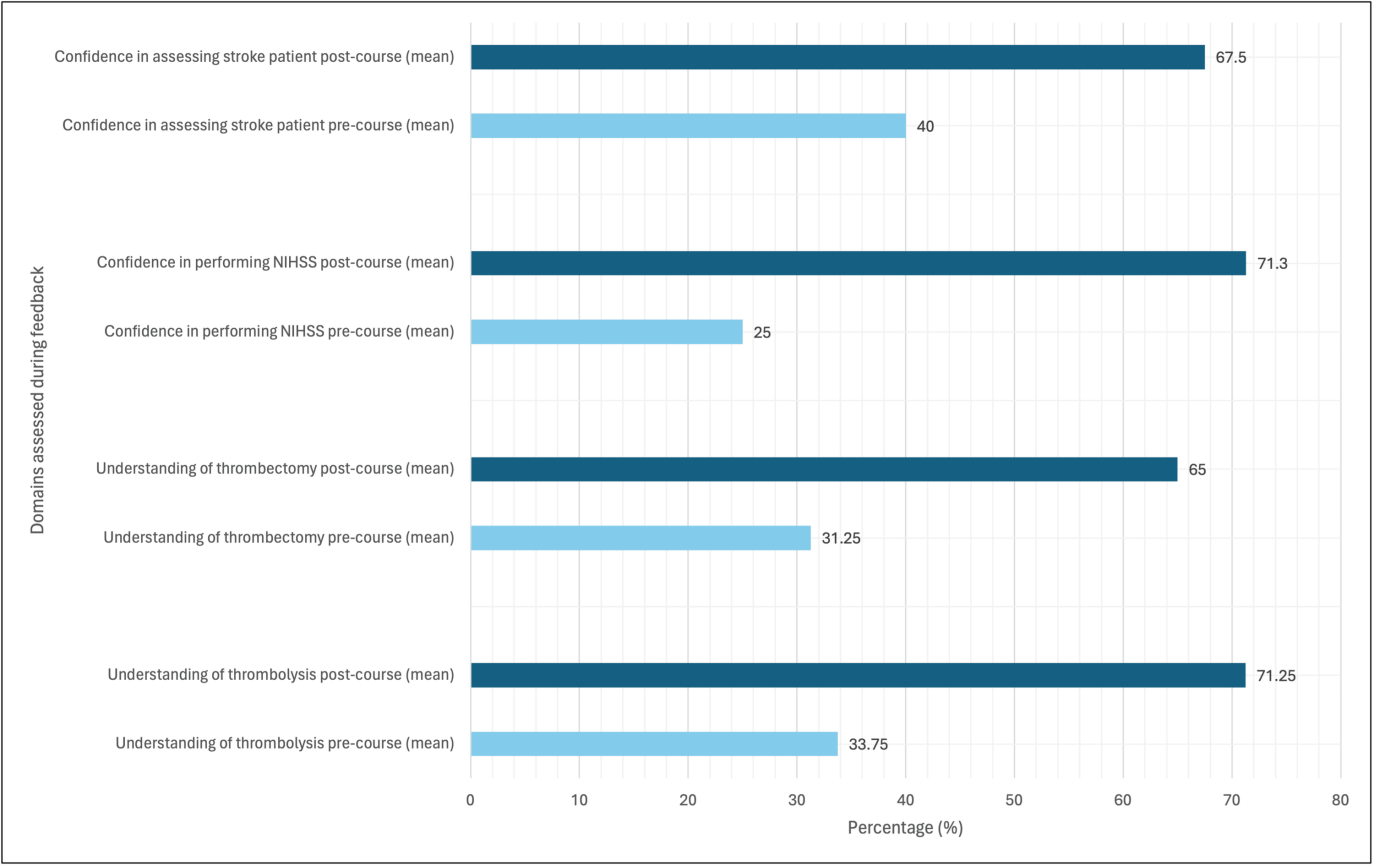
Summary of pre- and post-teaching questionnaire feedback

There was a significant increase by a mean of 2.75 (95% CI: 1.51-3.99) in understanding the assessment of stroke (p=0.0012). Confidence in completing NIHSS scoring increased by a mean of 4.33 (95% CI: 3.55-5.12), which was a significant finding (p<0.0001). The mean difference in improvement of understanding thrombolysis was significant (p=0.0003) with a mean difference in score of 3.75 (95% CI: 2.43-5.07). Similarly, understanding thrombectomy significantly improved (p=0.0002) with a mean difference in score of 3.4 (95% CI: 2.28-4.46). The results are illustrated as percentages in Figure 1.

**Figure 2:**
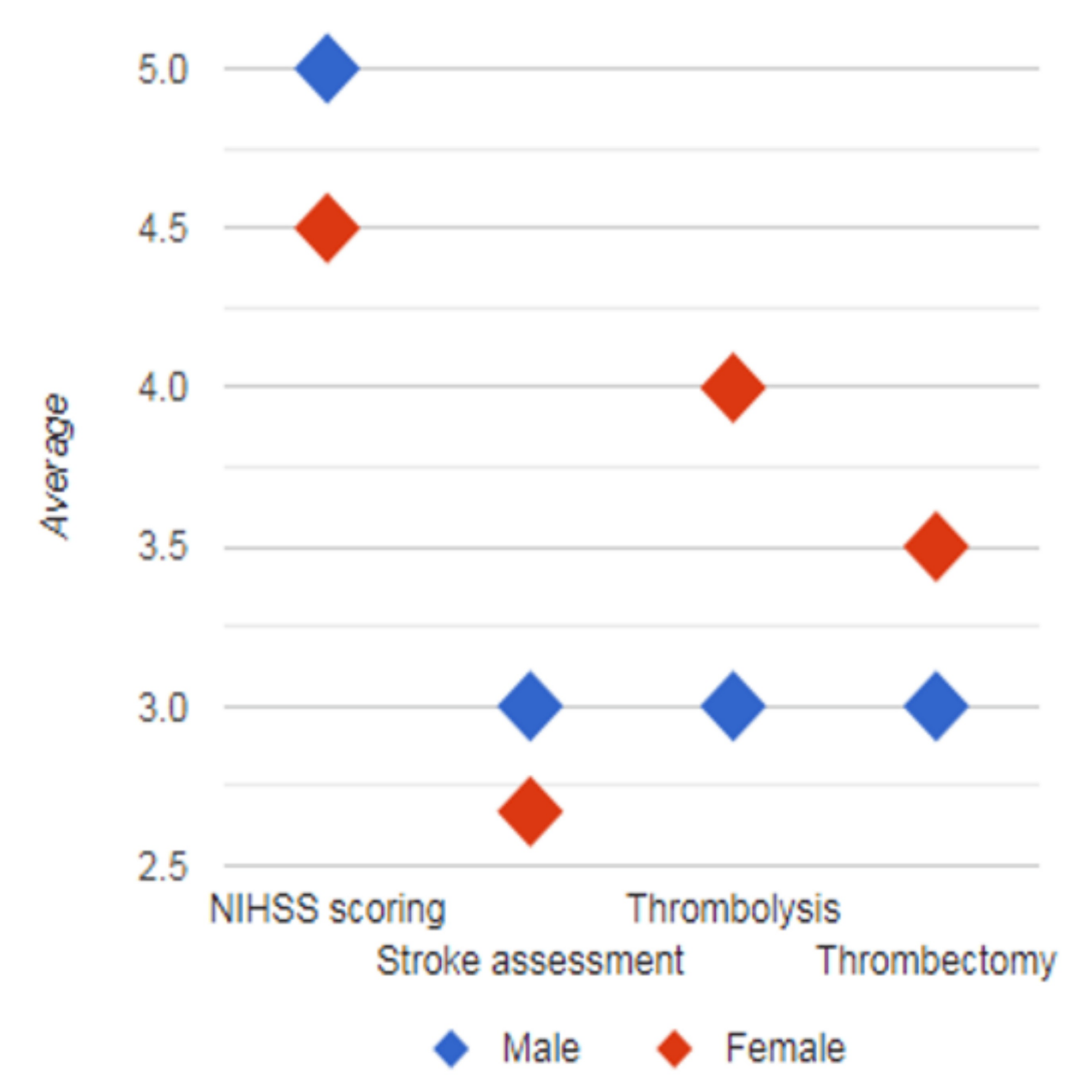
Summary of improvement in mean difference of scores by gender The X-axis represents domains (understanding thrombolysis, understanding thrombectomy, confidence in performing NIHSS, and confidence in the assessment of stroke patients), and the Y-axis shows the mean score difference.

**Figure 3:**
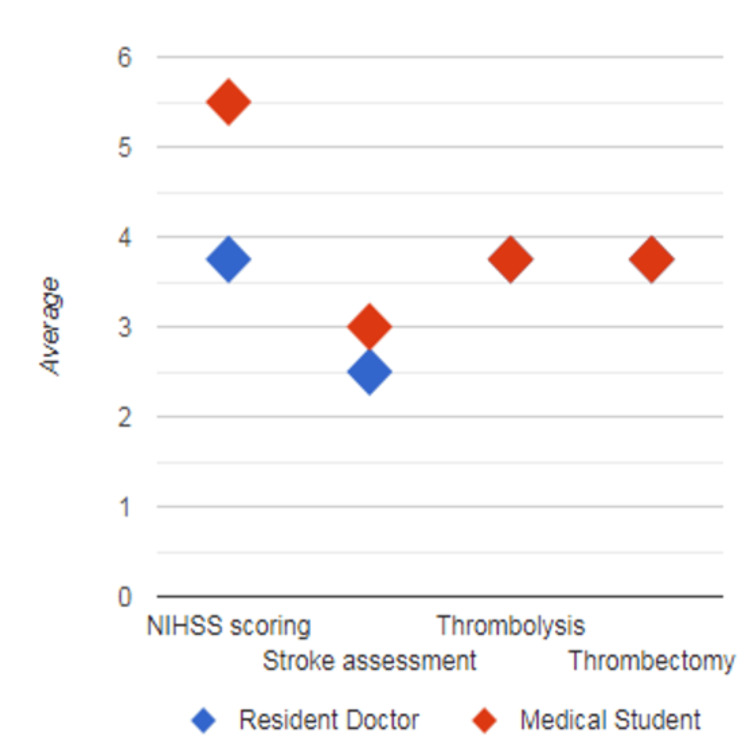
Summary of improvement in mean difference of score by age and experience (medical students <24 years old vs resident doctors >24 years old) The X-axis represents domains (understanding thrombolysis, understanding thrombectomy, confidence in performing NIHSS, and confidence in the assessment of stroke patients), and the Y-axis shows the mean score difference.

Further subgroup analysis showed that female participants had a greater improvement in mean difference compared to male participants in domains of understanding thrombolysis and understanding thrombectomy, whereas male participants had a greater difference in score in domains of confidence in performing the NIHSS and confidence in assessment of stroke patients, respectively (p=0.668; Figure 2). Medical students (<24 years) also showed greater improvement of mean difference in score compared to resident doctors (>24 years) in domains of confidence in performing NIHSS and confidence in the assessment of stroke patients; however, both showed equivocal improvement based on mean difference in domains of understanding thrombolysis and understanding thrombectomy, respectively (p=0.267; Figure 3).

The qualitative analysis from the post-course primary survey is displayed in Table 2. Most of the participants (96.8%) have not attended a stroke simulation course previously. Of 24 participants, 95.8% found the course at least good, if not very good or excellent, and 91.7% would recommend this course to others. 

**Table 2:** Summary of qualitative feedback

Category	Comments (n)			
Overall course rating	Excellent (9)	Very good (12)	Good (2)	Fair (1)
Recommend course to others	Yes (22)	Maybe (2)		

## Discussion

Simulation has been known to have poor uptake in neurology-based teaching [15], which is consistent with our findings that 96.8% of participants have never been to a neurology-based simulation previously. However, simulation has shown promise of reducing DTN time by approximately 15 minutes [12], which in turn reduces inpatient mortality by 5% [16] and improves outcomes by 4% [17]. Hence, we adopted a hybrid approach of both traditional teaching and simulation-based teaching to best equip participants to perform a focused clinical assessment of patients presenting with potential stroke. Pre-course materials covered basic concepts of stroke, including clinical recognition, Bamford and Trial of Org 10172 in Acute Stroke Treatment (TOAST) classifications and lectures to ensure a basic understanding of stroke prior to attendance. Our demonstration of NIHSS scoring, which was assessed during simulation, contributed to the highest improvement post-course with a mean increase of 4.33 (95% CI: 3.55-5.12, p<0.0001). 

The understanding of both indications for thrombolysis and thrombectomy was generally poor according to self-assessment ratings by candidates at 3.38 points and 3.13 points, respectively, pre-attendance. Our theory lecture ensured we improved baseline knowledge, which was reflected in a mean improvement of 3.75 points in the understanding of thrombolysis (p=0.0003) and a mean improvement of 3.375 points in the understanding of thrombectomy (p=0.0002), respectively. A meta-analysis of two studies showed a statistically significant effect favouring post-simulation training with respect to improved feelings of safety in thrombolysis-related decision-making, with a pooled risk ratio (RR) of 0.46 (95% CI: 0.36-0.59) [12]. An increase in knowledge can result in improvement in anticipating thrombolysis versus thrombectomy and awareness for appropriate brain imaging, which should be completed within one hour of arrival as per the National Clinical Guideline for Stroke 2023 (which is National Institute for Health and Care Excellence (NICE) accredited) [18]. This would ensure clear indications for CT or MRI of the head with appropriate stroke protocols that can be vetted by radiologists faster, given the indication is clear as per the new guidance [18].

Stroke simulation also provides a safe space for teams to test their knowledge and emulate the teamwork structure of the acute thrombolysis team with a designated thrombolysis nurse, resident doctor and a stroke specialist, underscoring the importance of interprofessional communication in thrombolysis calls [14,19]. Our course was unique since we relied on our participants in small group teaching to play different roles in pre-alert scenarios wherein one participant was the attending clinician to assess and treat a patient played by a course organiser. This provided additional insight into non-technical skills to participants, such as the challenges of prioritisation as a thrombolysis nurse with several emergency calls or being the stroke specialist with decision responsibility. All participants had assessed an unwell patient at least once in the acute scenarios, leading to an increase in knowledge, evidenced by a mean improvement of 2.75 points (p=0.0012) in confidence in assessing stroke patients. A meta-analysis of four studies showed similar statistically significant findings in favour of post-simulation training in improving healthcare professionals' acute stroke knowledge, with a pooled RR of 0.42 (95% CI: 0.30-0.60) [12]. 

Interestingly, our study found that female participants had a greater improvement in mean difference compared to male participants in domains of understanding thrombolysis and understanding thrombectomy, whereas male participants had a greater difference in score in domains of confidence in performing the NIHSS and confidence in the assessment of stroke patients, respectively (p=0.668). A larger sample size will be required for further study before conclusive remarks can be made. Our study also demonstrates that medical students (<24 years old) showed greater improvement of mean difference in score compared to resident doctors (>24y) in domains of confidence in performing the NIHSS and confidence in the assessment of stroke patients; however, both showed equivocal improvement based on mean difference in domains of understanding thrombolysis and understanding thrombectomy, respectively (p=0.267). This suggests that medical students may be less experienced and have the greater benefit of learning to assess stroke patients and perform NIHSS scoring, which may have been exposed to resident doctors, yielding a slightly greater improvement. We also suspect there were equivocal improvement rates in understanding thrombolysis and thrombectomy as this is specialist knowledge, which new resident doctors and medical students equally lack exposure to, resulting in similar levels of improvement post-course.

It is also important to consider that while reducing DTN improves outcomes for patients presenting with a stroke, a prospective observational study showed that reducing DTN was associated with a significant increase (15.9% vs. 24.4%, p=0.003) in the proportion of patients with stroke mimics (SM) receiving treatment. The study demonstrates that after the introduction of simulation training, 1.3% (p=1.0) of SM patients suffered asymptomatic intracerebral haemorrhage (ICH) vs 0% prior to simulation training [20]. Another retrospective observational study found that no SM patient had an ICH; three SM patients (17.6%) had systemic haemorrhages, two of which had gingival bleeding. The third systemic haemorrhage occurred in a patient who presented following a fall resulting in a spinal cord contusion and who, after intravenous tissue plasminogen activator (IV tPA) administration, developed radiographic evidence of a spinal epidural haematoma that required surgical evacuation [21]. Hence, simulation training has the potential for negative consequences such as an increased risk of bleeding in SM patients, increased use of resources, and, therefore, increased costs.

Limitations of the study include the fact that DTN time was not formally measured pre and post-simulation; hence, we cannot conclusively confirm our course-reduced DTN. Furthermore, although simulation is meant to be comparable to real-world environments, we have not formally assessed our course participants' performance in the real world to conclude transferability. An enhanced questionnaire was introduced midway through our course, which included further feedback collected on understanding thrombolysis, understanding thrombectomy, and confidence in the assessment of stroke patients. Furthermore, not all participants had completed pre- and post-course questionnaires. This reduced our sample size in domains of understanding thrombolysis, understanding thrombectomy, and confidence in the assessment of stroke patients to eight participants. We also note that our course participants included medical students and resident doctors of various grades who have differing preliminary knowledge; hence, this could overestimate or underestimate the improvement. Our results are generalisable to resident doctors and medical students. 

## Conclusions

Conducting a stroke simulation course has a positive impact on attendees in terms of improving clinical confidence with the assessment of stroke patients. Students were additionally informed about an online NIHSS course they could complete on their own to further improve their skills. Few participants have never attended stroke simulation training before, further emphasizing the value of embedding similar courses in medical education and initial years of training as resident doctors, thus improving the quality of care. Further research with a larger sample size needs to be conducted comparing DTN time pre- and post-simulation, and follow-up is required in real-world scenarios to assess transferability. This study validates our course as a success in improving understanding of thrombolysis, understanding of thrombectomy, confidence in performing NIHSS, and confidence in assessment of stroke patients.

## Appendices

Appendix A: Stroke simulation training pre-course questionnaire

Email: 

1. Have you ever participated in any stroke simulation training before? 

Yes 

No

2. If you answered yes to the previous question, please share your experience. Otherwise, please answer with N/A.

3. Have you ever attended any stroke pre-alert before?

Yes 

No

4. If you answered yes to the previous question, please describe how it went. If not, please answer with N/A.

5. Have you ever attended or done a NIHSS course (face-to-face/online)?

Yes

No

6. How confident are you in doing NIHSS scoring? 

Scoring scale from 1-10:

1 - Not very confident 

10 - Very confident 

7. How confident are you in assessing stroke patient? 

Scoring scale from 1-10:

1 - Not very confident

10 - Very confident 

8. How would you rate your understanding of thrombolysis? 

Scoring scale from 1-10:

1 - Not very confident 

10 - Very confident 

9. How would you rate your understanding of thrombectomy? 

Scoring scale from 1-10:

1- Not very confident 

10 - Very confident 

10. How would you rate your understanding of haemorrhagic stroke and principles of management?

Scoring scale from 1-10:

1 - Not very confident 

10 - Very confident 

11. Do you know where you can find the stroke guidelines and who to contact during business hours and out of hours?

Appendix B: Stroke simulation training post-course questionnaire

Email: 

1. How confident are you now in doing the NIHSS score?

Scoring scale from 1-10:

1 - Not very confident 

10 - Very confident 

2. How would you rate your understanding of thrombolysis?

Scoring scale from 1-10:

1 - Not very confident 

10 - Very confident 

3. How confident are you now in assessing a stroke patient?

Scoring scale from 1-10:

1 - Not very confident 

10 - Very confident 

4. How confident are you now in managing a stroke patient?

Scoring scale from 1-10:

1 - Not very confident 

10 - Very confident 

5. How would you rate your understanding of thrombectomy? 

Scoring scale from 1-10:

1 - Not very confident 

10 - Very confident 

6. How useful was the pre-course booklet prior to attending the course?

Scoring scale from 1-10:

1 - Not very useful

10 - Very useful

7. How would you rate the course overall?

Excellent

Very good

Good

Fair 

Poor

8. Would you recommend this course to others?

Yes

No

Maybe

9. What went well and what did you learn?

10. What could be done to improve the course?
